# SALIVARY CORTISOL AND METABOLIC SYNDROME COMPONENT’S ASSOCIATION

**DOI:** 10.1590/0102-672020180001e1351

**Published:** 2018-06-21

**Authors:** Grasiane Izidorio GARBELLOTTO, Fernanda Jardim REIS, Ana Maria Pandolfo FEOLI, Carla Haas PIOVESAN, Andréia da Silva GUSTAVO, Margareth da Silva OLIVEIRA, Fabrício Edler MACAGNAN, Carlos Alexandre Sanchez FERREIRA, Moisés Evandro BAUER, Cácio Ricardo WIETZYCOSKI

**Affiliations:** 1 Faculty of Nursing, Nutrition and Physiotherapy, Pontifícia Universidade Católica do Rio Grande do Sul; 2 Post-graduate Programa in Molecular and Cellular Biology, Biosciences Faculty, PUCRS, Porto Alegre, RS, Brazil.

**Keywords:** Obesity, Metabolic syndrome, Hydrocortisone., Obesidade, Síndrome metabólica, Hidrocortisona.

## Abstract

**Background:**

Actually the lifestyle exposes the population to several risk factors related to alimentary habits and less physical activity that contributes to chronic diseases appearance worldwide.

**Aim:**

To analyze the association between salivary cortisol and the components of metabolic syndrome.

**Methods:**

This is a cross-sectional study. As part of it, 28 individuals aged 30-59 years presenting three or more of the following findings: CA: ≥88 cm for women and ≥102 cm for men; SBP>130 mmHg and DBP>85 mmHg; GL>100 mg/dl; TG>150 mg/dl; HDL<40 mg/dl for men and <50 mg/dl for women. Was performed analysis of salivary cortisol (by radioimmunoassay) from 25 salivary samples collected throughout the day, for evaluating changes in the circadian rhythm of this hormone (8AM, noon and 8PM).

**Results:**

28 evaluated individuals had a mean age of 51.9±7.5 years, mostly women (64.3%) and a mean of BMI 33.6±3.2 kg/m². The cortisol level from the 8AM averaged 18.7±4.8 ng/dlL. Individuals with FPG>110mg/dl, have significantly lower average levels of cortisol than ones with FPG <110 (12.8±5,2 vs. 17.3±4.2). Significant correlations were HOMA vs. WC (r=0,465; p**˂**0,005) and TG (r=0,473; p**˂**0,005), WC vs. FG (r=0,446; p**˂**0,005) and BMI (r=0,730; p**˂**0.0001); TG vs. HDL (r=0,441 p**˂**0,005) and FG (r=0,440; p**˂**0,005).

**Conclusion:**

Morning salivary cortisol in subjects with chronically elevated blood glucose can represent a downregulation of the hypothalamic-pituitary adrenal axis. This is an important finding not yet well investigated.

## RESUMO

**Racional:**

Atualmente o estilo de vida expõe a população a diversos fatores de risco relacionados a hábitos alimentares e à inatividade física, contribuindo para o surgimento de doenças crônicas.

**Objetivo:**

Analisar a associação entre o cortisol salivar e os componentes da síndrome metabólica.

**Métodos:**

Estudo transversal com 28 indivíduos, idade entre 30 e 59 anos apresentando três ou mais dos seguintes achados: circunferência abdominal ≥88 cm (mulheres) e ≥102 cm (homens); pressão arterial sistólica >130 mmHg e pressão arterial diastólica >85 mmHg; glicemia >100 mg/dl; triglicerídeo >150 mg/dl; lipoproteína de alta densidade <40 mg/dl (homens) e <50 mg/dl (mulheres). Foram realizadas coletas do cortisol salivar nos seguintes horários 8 h, 12 h e 20 h e analisadas por radioimunoensaio.

**Resultados:**

A média de idade foi 51,9±7,5 anos, 64,3% eram mulheres e a média do índice de massa corporal foi 33,6±3,2 kg/m². O nível de cortisol salivar às 8 h teve média de 18,7±4,8 ng/dl. Os indivíduos com glicemia de jejum >110 mg/dl, apresentaram níveis médios de cortisol significativamente menores que os com glicemia de jejum <110 mg/dl (12,8±5,2 vs. 17,3±4,2). As correlações significativas foram HOMA vs. circunferência abdominal (r=0,465; p˂0,005) e triglicerídeos (r=0,473; p˂0,005), circunferência abdominal vs. glicemia de jejum (r=0,446; p˂0,005) e índice de massa corporal (r=0,730; p˂0,0001), triglicerídeos vs. lipoproteína de alta densidade (r=0,441 p˂0,005) e glicemia de jejum (r=0,440; p˂0,005).

**Conclusão:**

O cortisol salivar pela manhã, em indivíduos com glicemia cronicamente elevada, pode representar uma contraregulação do eixo hipotálamo-hipófise-adrenal, sendo achado importante e pouco investigado.

## INTRODUCTION

The current lifestyle exposes the population to several risk factors related to eating habits and lack of physical activity, contributing to the emergence of chronic diseases. Among these, cardiovascular, diabetes and obesity have become common causes of premature death^18^. Cardiovascular disease is the final event of several systemic alterations caused by other diseases such as hypertension, obesity, diabetes and hypercholesterolemia, which make up the so-called metabolic syndrome (MS)^9^ ^,^ ^17^. It is a complex set of cardiovascular risk factors, related to abdominal fat and insulin resistance, with high cardiovascular morbidity and mortality^9^ ^,^ ^17^.

According to The Third Report of the National Cholesterol Education Program - NCEP-ATP III15, diagnosis of MS is necessary to alter three of the five risk factors: abdominal obesity; abdominal circumference ≤102 cm (men) or ≤88 cm (women); triglycerides (TGL) ≥150 mg/dl; HDL<40 mg/dl (men) or <50 mg/dl (women); systolic blood pressure ≥130/85 mmHg and fasting glycemia ≥100 mg/dl, regardless of the presence of diabetes^16^ ^,^ ^22^.

Another important metabolic marker is salivary cortisol, which serves as a barometer of stress^27^. It is synthesized from cholesterol and its production is stimulated by the adrenocorticotropic hormone (ACTH) which is regulated by the corticotropin releasing factor and excreted in urine, blood plasma and saliva. The action of cortisol affects several physiological systems such as immune function, glucose regulation, vascular tone, and bone metabolism. Its production has circadian rhythm that depends on ACTH, with maximum levels in the morning decreasing throughout the day. ACTH and cortisol are secreted independently of the circadian rhythm as a reaction to physical and psychological stress^27^. In individuals exposed to constant chronic stressors, excess of cortisol is very harmful to health^11^.

The relationship between salivary cortisol and SM components evidences important stress response markers of the systemic inflammatory process associated with MS and its components.

The present study aims to demonstrate the results of the association between salivary cortisol and the components of MS.

## METHODS

All selected participants signed the Informed Consent Form and individually received information about the study procedures. The confidentiality and confidentiality requirements of the information collected were complied with in accordance with Resolution No. 466/2012, which establishes the guidelines for human research. The project was approved by the ethics committee of the institution, under number: 10/05153. The main study was registered in the Brazilian Registry of Clinical Trials, ReBEC, under number: RBR-9wz5fc. The authors committed themselves to maintaining the confidentiality of the data.

This is a cross-sectional study based on secondary data extracted from the study on the effects of different lifestyle modification interventions on physical, metabolic and behavioral aspects involved in the metabolic syndrome through the MERC (Research Modification Style Group of Life and Cardiovascular Risk of Catholic University, RS, Brazil). From this study, 28 gender-independent individuals, aged 30-59 years, were analyzed. As an inclusion criterion, they had to present three or more of the following findings that characterize SM^7^: abdominal circumference of ≥88 cm for women and ≥102 cm for men; systolic blood pressure >130 mmHg and diastolic >85mmHg; fasting glucose >100mg/dl; triglycerides >150mg/dl; HDL cholesterol <40 mg/dl for men and <50 mg/dl for women. Those who did not present all the necessary records in the main study database were excluded.

The assessment included the measurement of body weight, height and waist circumference. The body weight was verified by means of a scale with capacity for 160 kg, duly calibrated, with the patient barefoot and with the least possible clothing. A vertical anthropometer was used to measure height. Abdominal circumference was assessed through the abdominal perimeter (cm), measured at the site of maximum extension of the abdominal region^12^. The instrument used was an inelastic millimeter tape measuring 180 cm in length.

The values ​​of systolic and diastolic blood pressure were measured according to the recommendations of V Brazilian Guideline for Hypertension^24^.

For blood collection, volunteers were previously instructed to fast for 10-12 h. The collection of saliva was performed by the volunteer for later delivery to the research group. The evaluation of salivary cortisol was measured from samples collected throughout the day to evaluate changes in the circadian rhythm of this hormone, being 8 h (C8), at noon (C12) and at 20 h (C20). All collections were done before meals. They were used small rolls of cotton, previously prepared and sterilized for individual use. As for the method of collection the participant was advised to put the cotton roll under the tongue and leave for 3 min until it became saturated. Then remove the cotton and put it inside a 5 ml syringe, without the plunger. After replacing the plunger in the syringe, they were oriented to press the cotton, collecting the saliva in a plastic tube properly identified with the study number, name and time of collection. The minimum volume of saliva collected for each hour was 0.5 ml. The samples were centrifuged for 5 min at 1000 rpm and kept in a freezer (-20° C) until analysis, performed by radio immunoassay with a high sensitivity of 0.09 ng/ml (DBC - Diagnostics Biochem Canada)^3^ ^,^ ^10^.

The blood biochemical markers analyzed were: lipid profile (HDL-c, total cholesterol (CT), triglycerides (TGC) whereas the determination of LDL-c was performed indirectly - LDL-c=(TGC/5 + HDL)/CT), glycemic profile (fasting glucose, fasting insulin and HOMA). For the analysis of biochemical markers, 10 ml of blood were collected from each participant before and after the intervention, totaling 20 ml. Serum and plasma aliquots were separated for freezer storage at -80° C.

The IMC² was calculated according to the World Health Organization’s recommendation^8^ for nutritional status evaluation. The cutoff points for the classification of the individuals regarding BMI (kg/m²) were: normal 25 kg/m²; overweight from 25-29.99 kg/m²; obese ≥30 kg/m².

### Statistical analysis

The Kolmogorov-Smirnov test was used to verify the normal distribution of the continuous data. The data were presented quantitatively (mean±standard deviation). To determine the relationship between continuous variables, the Pearson and Chi-square correlation test were used to measure the association between categorical/dichotomous variables. Student’s T test was used to evaluate the difference between means. The level of significance was set at p=0.05 (bi-flow). For the analysis of the data the SPSS statistical package, version 22.0.0.0 was used.

## RESULTS

We evaluated 28 individuals with MS, with a mean age of 51.9±7.5 years; The majority were female (64.3%) with an average BMI of 33.66±3.27 kg/m².

**FIGURE 1:**
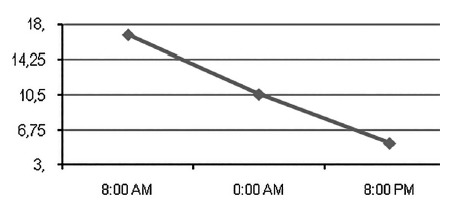
Salivary cortisol levels (ng/dl) in the 24 h period

The 8 h salivary cortisol level had a mean of 16.70±4.87 ng/ml; in the 12 h it was of 10.19±4.23 ng/ml; and in 20 h of 4.74±2.30 ng/ml. The cortisol drop throughout the day can be analyzed in Figure 1. The reference value of the 8 h salivary cortisol used was 13.5-23.5 ng/ml.

The data results of the patients and variables studied can be seen in Table 1.

**TABLE 1:** Characteristics of the sample

Variables	Mean±SD(n=28)
Age	51.86±7.46
Weight (kg)	91.72±12.34
Body mass index (kg/m²)	33.65 ±3.27
Abdominal circumference (cm)	109.04±8.39
Triglycerides (mg/dl)	196.86±94.71
High density lipoprotein - HDL (mg/dl)	47.29±11.73
Glucose (mg/dl)	107.57±36.42
Systolic blood pressure (mmHg)	133.68±16.06
Diastolic blood pressure (mmHg)	91.36±13.05

The results regarding the correlation between salivary cortisol and the components of SM can be seen in Table 2.

**TABLE 2:** Correlation between salivary cortisol and components of metabolic syndrome

Variables	Salivary cortisol					
	8 AM		Noon		8 PM	
	r	p*	r	p*	r	p*
Abdominal circumference	-0.133	0.499	-0.097	0.624	0.037	0.852
Systolic blood pressure	0.108	0.583	-0.140	0.478	-0.186	0.344
Diastolic blood pressure	0.000	0.997	-0.181	0.356	-0.264	0.174
High density lipoprotein - HDL	-0.191	0.331	-0.090	0.647	-0.224	0.252
Triglycerides	0.264	0.175	0.054	0.786	0.006	0.997
Glucose	0.052	0.792	-0.203	0.301	-0.197	0.314
Body mass index	-0.117	0.552	-0.043	0.829	0.048	0.808

*Pearson correlation

**FIGURE 2:**
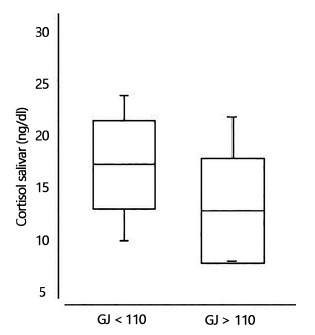
Relationship of morning cortisol to fasting glycemia (Boxplot chart showing mean±SD, p<0.05)

Figure 2 shows the division of patients regarding the presence or absence of each component of MS; in this way, individuals with fasting glycemia >110 mg/dl had significantly lower mean cortisol levels than those with fasting glycemia <110 mg/dl.

GJ=fasting glycemia

Insulin and HOMA measurements were available only in a subset of the study (n=24). Table 3 shows the various variables and correlations between the components of SM, HOMA and morning salivary cortisol.

**TABLE 3:** Correlation between the components of Metabolic Syndrome and Homestatic Model Assessment (HOMA)

HOMA	Total sample	r	p
Cortisol	24	-0.049	0.818
Abdominal circumference	24	0.465	0.022
Triglycerides	24	0.473	0.019
Insulin	24	0910	<0.001
Fasting glycemia	24	0.678	<0.001
Abdominal circumference	Total sample	r	p
Fasting glycemia	28	0.446	0.017
Diastolic blood pressure	28	0.375	0.050
Body mass index	28	0.730	<0.001
Triglycerides	Total sample	r	p
High density lipoprotein - HDL	28	0.441	0.019
Fasting glycemia	28	0.440	0.019

## DISCUSSION

When comparing the components of MS with salivary cortisol levels at 8AM, noon and 20PM, there is an important relationship between fasting glycemia and salivary cortisol. Individuals with fasting glycemia >110 mg/dl have significantly lower salivary cortisol levels than those with glycemia ˂110 mg/dl^2^.

Currently, the relation of the hypothalamic-pituitary-adrenal axis and alterations in glucose metabolism has been the subject of clinical studies^5^ ^,^ ^6^ ^,^ ^14^ ^,^ ^20^ ^,^ ^21^ ^,^ ^25^. A review of the recent literature has evaluated six studies that correlated morning cortisol with glucose; of these, only three demonstrated a positive association. Significantly higher levels of salivary cortisol at 10 h and dexamethasone-stimulated cortisol were found in subjects with type 2 diabetes mellitus when compared to the normoglycemic individuals. Salivary cortisol at 10 h correlated significantly with fasting glycemia and glycated hemoglobin. This study also demonstrated that diabetic subjects had a higher level of stress and stress response than non-diabetics, as measured by standard questionnaires. It is noteworthy that this study compared individuals with normal glycemic disorders with diabetics of recent diagnosis, and it can be inferred that the situation of significant stress due to the recent diagnosis of diabetes may have led to the increase of cortisol as a reaction to stress and not as a result of high blood glucose^23^.

Considering the results found in this study it can be inferred that the result of the lower salivary cortisol in subjects with fasting glycemia >110mg/dl observed in this study may be a paradoxical effect. This paradox could be explained by the fact that individuals presented chronic high fasting glycemia, and the acute stress period has already been overcome, and there is a possibility of downregulation of cortisol levels due to chronically high glycemia. That is, elevated insulin resistance, increased glycemia, and chronic oxidative stress counteract the adrenal pituitary hypothalamus axis in order to decrease morning cortisol levels in an attempt to return to metabolic homeostasis.

SM is a cluster of metabolic abnormalities that increases the risk of type 2 diabetes mellitus and cardiovascular disease, which can be defined as a state of metabolic homeostasis disorder characterized by a combination of central obesity, insulin resistance, dyslipidemia and hypertension^19^.

Salivary cortisol is an important marker of chronic stress and one of several stressors that can lead to changes in the hypothalamus adrenal pituitary axis, with alteration of the metabolic homeostasis of the organism and consequently to the metabolic disorders mentioned above^1^ ^,^ ^5^ ^,^ ^23^. There is strong evidence to support the hypothesis that glucocorticoids released in response to chronic stress induce accumulation of visceral fat that, when present in some conditions, such as excessive energy intake, low physical activity and poor quality of food, will trigger the mechanism development of SM^19^.

Although previous studies have clearly demonstrated the usefulness of salivary cortisol to assess the rate and activity of the hypothalamic pituitary adrenal axis and also for the diagnosis of hypercortisolism^13^, its clinical value as a marker of stress and metabolic changes in the body is not yet clearly defined.

The role of cortisol in the regulation of metabolic homeostasis is even controversial, while some articles show that it increases in individuals with MS^1^ ^,^ ^2^ ^,^ ^23^, a large study of 726 adults found no significant association between the components of MS and changes in levels of salivary cortisol throughout the day. However, individuals with MS had 16% lower salivary cortisol than those without this diagnosis^4^.

The dysfunction of the adrenal pituitary hypothalamus axis is associated with obesity and MS and, consequently, BMI; increased abdominal circumference and visceral fat are associated with low levels of salivary cortisol in the morning^19^. In this study, the relationship between the components of MS and their association with insulin resistance can be seen in Table 3. Although no significant correlation between insulin resistance measured by HOMA-IR and morning cortisol has been demonstrated, other authors suggest that salivary cortisol tend to increase with high BMI and correlate with hip circumference in men and with systolic pressure in women significantly. Even so, the study concluded that these data do not support a strong relationship between cortisol levels and SM components^1^.

Analyzing the relationship between salivary cortisol and obesity, a study of 82 subjects showed that there was no difference in the level of morning salivary cortisol between non-obese, obese and weight-loss individuals^2^. However, men who lost weight have a lower level than non-obese men; this relationship in women was not observed. The observation that visceral fat loss may reduce hypothalamic-pituitary-adrenal axis activity contrasts with the findings that weight loss associated with restriction of caloric intake increases the activity of the hypothalamic-pituitary axis^26^. From this it can be inferred that fasting and weight loss may increase circulating levels of cortisol, possibly to increase appetite in an attempt to “defend” itself from loss of body mass.

In fact, current literature shows inconsistent relationships between cortisol levels and metabolic parameters. While one study shows a tendency to increase salivary cortisol in higher BMI^1^, another shows an inverse correlation between morning salivary cortisol levels^2^. In this study, the correlation between cortisol and BMI was not significant, most probably because the sample did not have patients with very high BMI (no patient with BMI >40 kg/m²).

Previous studies have reported that individual components of MS and activity of the hypothalamic-pituitary-adrenal axis are related; however, few studies have examined the components individually. Specifically, elevated hip circumference has been associated with high rates of morning cortisol and related to stress reactivity as well as decreasing cortisol variability throughout the day. Evidence suggests that hypertension is associated with morning cortisol levels and/or cortisol stress reactivity. Studies of the BMI association and central adiposity with cortisol have been misleading with some reporting that people with high BMI and adiposity rates have high cortisol under-curves; but others report that people with high BMI and waist circumference have low levels of cortisol at dawn and a decrease in the rate of daily drop in cortisol levels^1^ ^,^ ^4^.

## CONCLUSIONS

Salivary cortisol in the morning in individuals with chronically elevated glycemia may represent a counter-regulation of the hypothalamic-pituitary-adrenal axis, being important marker and not well investigated till now.
